# Barriers general education teachers face regarding the inclusion of students with autism

**DOI:** 10.3389/fpsyg.2022.873248

**Published:** 2022-08-22

**Authors:** Mohammed Al Jaffal

**Affiliations:** Department of Special Education, College of Education, King Saud University, Riyadh, Saudi Arabia

**Keywords:** autism spectrum disorder, ASD, inclusive education, students, qualitative research, least restrictive environment, inclusion, general education teachers

## Abstract

As the number of students diagnosed with autism spectrum disorder (ASD) present in general education (GE) classrooms has increased in the past few decades, GE teachers must adapt to meet the needs of these students. Laws and regulations require students with ASD to be educated in the least restrictive environment, as well as that they be instructed by the teachers who were qualified to teach them. Unfortunately, GE teachers face the challenges supporting students with ASD in GE settings. This qualitative research investigates the barriers that prevent teachers from successfully implementing an inclusive environment in the GE classroom. In total, four elementary school teachers at a school in the northeast of the United States were interviewed and observed. The data were analyzed to identify emerging themes. The findings showed that GE teachers lack training in how to work with students with ASD in their GE classrooms, lack collaboration opportunities with their special education colleagues to better support their students with ASD, and are not provided sufficient resources by their schools and programs to create an appropriate inclusive environment in their GE classrooms. Based on these findings, certain improvements in professional development offerings for in-service general educators on how to teach students with ASD are recommended, such as providing broader training programs that give teachers the opportunity to practice interventions and teaching plans for inclusive classrooms and receive feedback from the training instructor(s). Furthermore, certain additions to the curriculum of pre-service university education programs for GE teachers are suggested. In addition, the research found that schools must make certain resources, including technology, available to GE teachers to meet the requirements of United States law regarding educating students with disabilities, including ASD, in the least restrictive environment, which is the GE classroom.

## Introduction

Autism spectrum disorder (ASD) is a neurodevelopmental disorder characterized by deficits in social communication and the presence of restricted interests and repetitive behaviors (American Psychiatric Association., 2013). Moreover, the condition may impact the educational performance of the child with ASD, due to the disorder being characterized by resistance to any change in the daily routine or environment, non-typical responses to all kinds of sensory experiences, and the engagement in activities of a repetitive nature [34 C.F.R. 300.8 (c) (1)]. The Individuals With Disabilities Education Act (Individuals with Disabilities Education Act [IDEA]., 2004) aimed to ensure that all children with disabilities have access to free and appropriate education in the least restrictive environment and are supported by related services designed to prepare them for the future. In addition, this legislation outlines the requirements for the assessment of these children. Furthermore, it ensures that educators have all the required tools to improve the education of students with disabilities (Trohanis, 2008; Yell, 2012). IDEA brought about significant changes to the nation’s school systems as a whole (Moores-Abdool, 2010). It also increased the number of students with ASD in general education (GE) classrooms.

Reporting conducted for the Centers for Disease Control and Prevention (CDC; Maenner et al., 2020) estimated that 1 in 54 children has been identified as having ASD in the United States. In addition, the number of individuals with ASD who are school-aged is about 1 in 50 (Blumberg et al., 2013). Many students with disabilities are now included in GE classes for most of the school day (National Center for Education Statistics., 2021). Goodman and Williams (2007) stated that “The increased numbers of students with ASD that educators encounter in mainstream settings result not only from legal and empirical support for this placement option but also from increases in the incidence of this disorder” (p. 53). Furthermore, the right of students with ASD to be educated with their typical peers in the GE classroom is endorsed by many teachers and parents (Hasson et al., 2022). Thus, in the present day, it is expected that general educators be capable of accommodating these students in their classrooms (Boyle et al., 2022). Unfortunately, despite these expectations and legislative requirements, few models and procedures have been developed to facilitate the successful placement and maintenance of students with ASD in GE classrooms (Moores-Abdool, 2010; Klibthong and Agbenyega, 2022).

Students with ASD face a range of challenges in the inclusive setting, involving social, academic, and behavioral issues (Allen and Yau, 2019). They struggle with social and communication skills delay as well as issues with play and learning (Mody and Belliveau, 2013); certain typical ASD behaviors might also affect the ability of these students to participate in classroom learning activities and engage with peers (Conallen and Reed, 2017). For example, individuals with ASD might experience language delay, but even more relevant in the classroom setting is that their difficulties with communication skill development can manifest as “social withdrawal” and a “lack of social reciprocity”—failure to engage in conversation and social interactions in a way that is typical—causing them to be isolated from their peers in the classroom during group activities or play (Mody and Belliveau, 2013, p. 159). Moreover, the verbal outbursts and resistant behavior that children with ASD might exhibit can cause disruptions in the GE classroom for teachers as well as draw negative reactions from the children’s typical peers (Mody and Belliveau, 2013).

Not all students with ASD are exactly the same, which creates additional challenges for teachers when these students are in GE classrooms (Finlay et al., 2022; Leonard and Smyth, 2022). Clearly, for students with ASD to receive appropriate education in GE classrooms, modifications to curricula and instruction are necessary; however, these can vary significantly depending on the level of functionality of the individual student (Moores-Abdool, 2010). Meeting the needs of these students presents unique challenges for teachers (Hodges et al., 2020). Moreover, this can be a daunting task without clear guidelines (Simpson et al., 2003; Van Der Steen et al., 2020). This study examined the barriers the GE teachers encounter when students with ASD are present in the GE classroom. In addition, it aimed to find out what GE teachers require to overcome the identified barriers and achieve successful inclusive classroom environments.

## Literature review

When students with ASD are included in the GE classroom, their GE teachers are responsible for addressing their needs, just as they are responsible for addressing the needs of their typical peers (Boyle et al., 2022). However, research has found that GE teachers lack the training and professional development to successfully create an inclusive setting (Gómez-Marí et al., 2021, 2022). Furthermore, teachers themselves have highlighted the need for better collaboration opportunities with special education professionals and more technology to meet the requirements of legislation such as IDEA (Mulholland and O’Connor, 2016).

### Pre-service training

Most universities only require that university students studying to become GE teachers take a few classes in special education; therefore, most teachers start their careers with less to no training in the field of autism and ASD (Morton and Campbell, 2008; Barned et al., 2011; Busby et al., 2012). This lack of training at the college level often leaves the teachers ill-equipped to effectively teach students with ASD (Suhrheinrich, 2011). Numerous studies have found that a lack of training at the university level is also a barrier to successful implementation of interventions (Morrier et al., 2011; Alexander et al., 2015). Moreover, even when teacher education programs have expanded their offerings regarding the education of those with disabilities over the years, they often still fall short of training GE teacher students to the specific degree needed (Scheuermann et al., 2003).

Barned et al. (2011) conducted a study to evaluate and assess pre-service early childhood education teachers regarding the inclusion of students with ASD that included conducting a survey with 15 such pre-service teachers at Southeastern University to assess their knowledge and attitudes toward the inclusion of students with autism. In addition to completing the survey, four of the teachers were also interviewed. The results showed that there is a lack of knowledge on the part of pre-service teachers regarding ASD, as well as certain misconceptions about the condition and the needs of students with ASD in inclusive classrooms. The participants showed an interest in obtaining a better understanding of ASD and demonstrated the support of the inclusive classroom. However, their attitudes toward the inclusion of students with severe disabilities were ambiguous. The findings from this study indicated that pre-service teacher preparation programs should be expanded to deliver additional knowledge about ASD and foster more inclusive attitudes toward these students (Barned et al., 2011). To add to this, general educators have expressed different insecurities about teaching children with autism due to feelings of inadequacy regarding their university education and training (Roberts and Webster, 2022). It is critical that this lack in teacher training be addressed, so that first-year teachers are adequately prepared to teach students with ASD (McCray and McHatton, 2011).

### Professional development

The research has consistently shown that ineffective professional development is a barrier to successful implementation of interventions for students with ASD in GE settings (Boyle et al., 2022). Although programs often provide training on interventions, there are many other obstacles that may limit effective implementation (Alexander et al., 2015). The traditional method of delivering professional development involves lectures and handouts that often prove to be ineffective as they fail to supply attendees (teachers) with actual methods for applying the information provided to the real word of the classroom (Bethune and Wood, 2013). Researchers also have found a need for more supportive professional development environments, such as ones that incorporate opportunities for teachers to practice the interventions they have learned during the sessions and obtain feedback from the instructor regarding their performance prior to being expected to successfully apply such practices in the classroom; this type of feedback as well as the provision of manuals and online support could increase the utilization of new instructional strategies in the classroom (Odom et al., 2010; Simonsen et al., 2010). Therefore, teachers may attempt to adapt these newly acquired skills to what they are already doing while neglecting the core components necessary to create an effective intervention (Odom et al., 2010). In addition, some educators may also be reluctant to try new interventions because they may not fit with the strategies they currently use (Lang et al., 2010). Odom et al. (2010) indicated that in the past, professional development often involved one-time workshops or presentations where no ongoing support was provided. This type of professional development has been shown to be ineffective in providing teachers with a full understanding of how to implement the program for the benefit of students (Chung et al., 2015; Bates and Morgan, 2018).

In a study by Brownell et al. (2013), it was found that educators often know more about the subjects they teach than the evidence-based practices (EBPs) that have been developed for creating effective instruction to teach those subjects. The ability to integrate new strategies and ideas into an easy-to-follow, comprehensible instructional approach has been found to be most influenced by the degree to which teachers analyze current practices and student needs and apply them to the content they are teaching (Brownell et al., 2013). The authors stated that “Teachers’ analysis seemed dependent on knowledge of special education practice and curriculum, knowledge for teaching reading, desire to learn, persistence in implementation, and use of PD (professional development) strategies to improve practice” (Brownell et al., 2013, p. 42).

### Teacher collaboration

It is not just pre-service teachers’ attitudes or their training as much as it is the structures of special and GE programs at the university level. Researchers assert that the reality of separate departments for special education and GE in teacher education programs contributes to the issues that arise in educating pre-service teachers how to collaborate to meet the needs of children with special needs (Silverman, 2007; Pülschen and Pülschen, 2015). For teachers to successfully implement interventions for students with ASD in the GE setting, collaboration between general and special education is essential (Simpson et al., 2003).

Instructing those with learning difficulties poses challenges for GE teachers, and it is important for them to have the ability to work with others within their schools with expertise in special education to enhance the inclusive environment of the GE classroom (Majoko, 2019). Due to the varying needs of children in the GE classroom, it is not possible for the general educator to work in isolation (Vakil et al., 2009). Without collaboration, it may be impossible to successfully implement certain interventions for students with ASD in the GE setting (Finlay et al., 2022).

A study was conducted by Able et al. (2015) to identify the needs of students with ASD in the GE classroom and the requirements of teachers to facilitate the success of their students. The researchers utilized two focus groups to explore and understand teacher preparation practices and teachers’ perspectives of the social support needs of students with ASD in the GE classroom. This study revealed that GE teachers indicate the need for more knowledge of ASD and of individualized instruction strategies for students with ASD in inclusive settings. They also emphasized the need for increased collaboration between general and special educators to include the students with ASD in GE settings (Able et al., 2015).

Although several studies have found that teachers hold positive attitudes toward students with ASD (e.g., Park and Chitiyo, 2011; Boyle et al., 2022), other research has found that teachers have negative perceptions of students with ASD, including that these students have low levels of academic achievement and emotional and social development (Gómez-Marí et al., 2021, 2022). As a result, teachers may be influenced by this prejudice toward students with ASD when such students are present in their classrooms, even if they do not realize it (Chung et al., 2015). Teachers have reported the feelings of frustration and guilt regarding the amount of time required to ensure that students with disabilities are accommodated, including the time required to modify lesson plans and curricula. They express concern regarding the perceived time such activities take away from students without disabilities who tend to comprise the majority of most classrooms (Lopes et al., 2004; Cassady, 2011).

## Materials and methods

### Setting and participants

This study took place in a private elementary school located in the northeast of the United States. This private school has a total enrollment of 120 students in grades pre-school through seventh grade and 20 GE teachers. The school does not have special education teachers, and some students with autism have enrolled at the school in the last 2 years. The students with ASD in the school are fully included in GE classrooms. The school community comprises low- to middle-income students of various ethnicities. Case studies, as Merriam (1988) described them, contain “an intensive, holistic description and analysis of a bounded phenomenon” (p. 8). Merriam also stated the main characteristic of a qualitative case study is that it presents a rich and in-depth description of the phenomenon or phenomena being investigated. Therefore, the private school that was the setting of this case study was chosen because the researcher had worked with staff there in the past to implement a positive behavioral interventions and supports (PBIS) program. Moreover, the researcher’s relationship with the staff made it possible to observe the phenomenon of the inclusion of students with ASD in the GE classroom in an in-depth manner. This program was also deemed to involve an appropriate population from whom to obtain qualitative data on the research topic, namely, GE teachers who have direct interactions with students with ASD in inclusive settings. In total, four of the 20 GE teachers at the school were recruited using a purposive sample of the educators to explore the phenomenon of the inclusion of students with ASD in the GE classroom and to obtain their insights into the barriers they encounter regarding teaching students with ASD in this setting. Purposive sampling method is commonly employed in qualitative research due to its usefulness in selecting individuals who possess the background and knowledge necessary to support the goals of the study and to provide the researcher with the ability to obtain the most appropriate sample to address the aims of the research (Marshall, 1996; Tashakkori and Teddlie, 2003).

In addition, all of these factors meet the requirements of Robinson (2014) regarding how to obtain an appropriate sample in qualitative research by ensuring that the population from which the sample is drawn is capable of providing insight and clarification of the identified phenomenon to be explored by the research. Inclusion criteria for participation in this study were as follows: general educators with at least 1 year of experience teaching students with ASD and who have students with ASD in their primary classroom. In addition, the teachers had to have at least a bachelor’s degree in education. Each teacher was sent the interview questions by an email a week before the interview to prepare for the questions. The teachers who participated in this study are designated as Teacher 1, Teacher 2, Teacher 3, and Teacher 4, to protect their privacy and for ease of reporting the data obtained from the interviews. Please refer Table 1 for the demographic information of the participants.

**TABLE 1:** Demographic data of the participants.

	Teacher’s role	Gender	Qualifications	Years of teaching	Years with students with ASD
1	General educator	Female	Bachelor’s degree in education	4	2
2	General educator	Female	Bachelor’s degree in education	6	2
3	General educator	Female	Bachelor’s degree in education	3	2
4	General educator	Female	Bachelor’s degree in education	4	2

### Study design and data collection

Qualitative research design was chosen for this study. Specifically, a case study involving teachers at a private school was conducted. The semi-structured interview questions were based on the review of the existing literature (Lindsay et al., 2013; Able et al., 2015; Van Der Steen et al., 2020) and designed to answer the research questions. The interview questions were reviewed by three professors with expertise in inclusive education and their suggestions were used to edit and improve the original questions. For instance, the reviewers suggested adding a question on the teachers’ beliefs regarding the inclusion of students with ASD, as this has been identified as a critical factor in overcoming barriers to successful inclusion of these students (refer to Appendix A for the interview questions). The interviews were audiotaped to allow the researcher to gather rich information about teachers’ experiences and opinions of the inclusion of students with ASD in GE classrooms in a format that could be referenced later. Each teacher was interviewed for 30–45 min to obtain an understanding of their barriers and needs regarding the inclusion of students with ASD in the GE classroom. To accommodate the participants’ schedules, two of the interviews were conducted in a private room at the school and the rest were conducted by cellphone. In addition to the interviews, the researcher used observation as another data collocation method. Thus, the four teachers were observed and notes were taken regarding how the individual teachers interacted with and instructed their students with ASD. The observations occurred over 1 day for each teacher.

### Data analysis

Data were analyzed based on the thematic analysis of what the participants said regarding the what, why, and how of their views about the specific topic. Following the thematic analysis, as proposed by Braun and Clarke (2006), data coding was accomplished by the researcher as follows. First, the audio recordings of the interviews were transcribed verbatim and the transcriptions were reviewed. As a part of this initial process, member check was performed by showing the transcription of their interview to each teacher interviewee, so that they could review it for accuracy and make any changes regarding misinterpretation, if needed. During the review of the transcriptions, the researcher’s notes from the interviews were also consulted so as to become familiarized with the data; notes were taken during this process to begin to identify themes. Next, all the data were coded to describe the content by highlighting and extracting key phrases from each of the interviews. After this, the researcher combined the similar coded phrases into a single theme; codes that only appeared once were excluded. This process involved thematic analysis using an indicative coding method that allows the researcher to generate themes and descriptors (Creswell, 2014). The data analysis also involved highlighting the themes and patterns and utilizing deductive coding to connect the findings to the literature review. The researcher also related the themes to the observations and the lesson plans to enhance the credibility of the data. Finally, the data were grouped and the themes were identified and conceptualized in relation to the research questions and the literature review. The observation and lesson plans supported the themes identified by the study. Additionally, double coding was conducted for each interview by a colleague of the researcher to review and confirm the coding forms. This individual is an associate professor with expertise in the field of special education.

## Findings

This research endeavored to identify the barriers the GE teachers’ state impact the teaching and inclusion of students with ASD in the GE setting. In addition, the study aimed to identify the tools these educators require to create a successful inclusive classroom environment for students with ASD. From the data obtained from the survey answers, interviews, and observations, three themes emerged:

1. Teachers’ lack of training regarding students with ASD.
2. Teachers’ lack of collaboration opportunities.
3. Lack of resources provided by schools.

Each of these themes is discussed in greater detail in the following sections (also please refer to Table 2).

**TABLE 2:** Presentation of themes and related recommendations.

Theme	Relevant quote(s)	Recommendation	Method(s) of implementing recommendation
Theme 1: Teachers’ lack of training regarding inclusion of students with ASD	Teacher 3: “I think definitely general education teachers need training because I believe that general education teachers are not prepared to deal with students with ASD.” Teacher 4: “Many courses must be taught to undergraduate students since they will be teachers in the future.”	Advocate for better training and preparation of general education teachers.	Implement expanded instruction at both the pre-service (during university programs) and in-service (professional development, school-provided program) levels.
Theme 2: Teachers’ lack of collaboration opportunities.	Teacher 2: “Collaboration is very important because it will help me to teach all the students.” Teacher 1: “It is very hard to work alone in the classroom.”	Advocate for greater collaboration between GE teachers and their colleagues with specialization in special education.	Implement at both the pre-service and in-service levels, by: (a) incorporating more SE curriculum into the course programming for GE teaching students; and (b) providing regular opportunities within the school week or month during which GE teachers and SE personnel can consult to discuss overall issues with students with disabilities, including ASD, as well as specific students with ASD.
Theme 3: Lack of resources provided by schools.	Teacher 4: “Lack of support and resources is a big issue.” Teacher 1: “Our private school does not receive fund form government it only depends on the enrollment and donation.”	Advocate for funding and specialized equipment.	School administrators/directors should seek additional funding from appropriate entities (or redirect existing funding) to support the necessary expansion of services to students with disabilities, including ASD, and to ensure the availability of necessary resources for these students through greater support for their GE teachers.

### Teachers’ lack of training regarding students with autism spectrum disorder

Teachers have reported the feelings of frustration and guilt over the amount of time required to accommodate and modify the lessons for students with autism (Cook and McDuffie-Landrum, 2020). Additionally, teachers state they feel this time takes away from the majority of their students who do not have disabilities (Lopes et al., 2004). It is not surprising teachers feel frustrated because they are unprepared to teach these students. Teachers in this study emphasized that training on how to teach students with ASD is important for providing an appropriate education for these students. In addition, they agreed that GE teachers are unprepared to teach students with ASD. For example, Teacher 1 stated that “I think that teachers who do not have any specialized background are unprepared (to teach students with ASD). I think GE teachers do not know how to adapt the curriculum or come up with a behavior management plan that is effective.” Teacher 3 stated that “I think definitely GE teachers need training because I believe that GE teachers are not prepared to deal with students with ASD.” The researcher’s observation data and the teachers’ lesson plans showed the teachers had difficulty addressing the needs of students with ASD. For instance, during one observation, a student with ASD was seen crying and trying to avoid doing a particular task. The teacher being observed was unable to implement any helpful strategy to support the student and chose to simply put the student in time out. It is notable that all the study participants showed positive attitudes toward the inclusion of students with ASD in the GE classroom, but only as long as they felt that they had been provided with the support necessary to be successful. For example, Teacher 3 stated that “Definitely, students with ASD should be included in the GE classroom because this is the real world. But I feel unprepared to work with students with ASD in the inclusive classroom with such minimal support.”

### Teachers’ lack of collaboration opportunities

To successfully include the students with ASD in the GE setting, collaboration between general and special education teachers is essential. To obtain positive results for all their students, it is critical that general educators have the opportunity to work with others (e.g., special education teachers, therapists, etc.) to enhance the inclusive environment of the classroom (Majoko, 2019). Teachers who participated in this study agreed that collaboration is essential when teaching students with disabilities in inclusive settings. Furthermore, they emphasized that they are not provided with the necessary support personnel to create a successful classroom environment when students with ASD are present in the GE classroom. For example, Teacher 2 stated the following:

I need special education teachers to collaborate with and help me to see what works for the student (with ASD) and what does not work for him, and also to see how my student can benefit (from different practices). I think special education teachers will definitely help, I think collaboration is very important because it will help me to teach all the students.

Similarly, Teacher 4 stated the following:

The minimum support I need is an aide, I need a person with me in the class for example, when I am teaching I need that person to help the student to open (the student with ASD’s) textbook to a certain page and tell the student what to do. It is very hard to work alone in class without collaborators.

Researcher observation supported that the teachers had difficulty in providing the needed level of support to the students with ASD in the classroom while also meeting the needs of their other students.

### Lack of resources in schools

Working with students with ASD in inclusive settings can be challenging for teachers; inadequate knowledge of ASD and the lack of access to support and the advice of professionals (e.g., therapists and paraprofessionals) with expertise in supporting these students only makes the job of the general educator even more difficult (de Boer, 2009). All teachers indicated that the lack of resources makes inclusion almost impossible to implement. For example, Teacher 3 said that, “The school has a lack of resources and this impacts students with disabilities in the school. Private schools do not receive funds from government. The only funds we receive are just for books and materials.” Furthermore, Teacher 4 stated the following:

We do not receive funds for hiring and training teachers. We depend on enrollment and donations. If you need to bring on a person as a counselor that will cost a lot, lack of support and resources is a big issue.

It was clear that the school lacks services and equipment to support students with ASD in the GE classroom. During the observation and the review of the lesson plans, it was also noted that the students at the school with ASD do not have Individualized Education Programs (IEPs). According to the teachers, they had submitted a formal request in writing to the public school district for support with evaluations and IEPs.

## Discussion

The research question that guided this study was, “What barriers are encountered by GE teachers when teaching students with ASD?” In addition, the purpose of the study was to find out what GE teachers need to overcome these barriers and successfully teach students with ASD in the inclusive classroom. The results of this study were consistent with those of similar studies (e.g., Lindsay et al., 2013; Hsiao and Sorensen Petersen, 2019; Van Der Steen et al., 2020) that found general educators need more knowledge, collaboration opportunities, and resources to support students with ASD in GE classrooms. In total, three main themes emerged from this qualitative study: teachers’ lack of training for students with ASD, teachers’ lack of collaboration opportunities, and the lack of resources in schools.

### Examination of the themes

First, the teachers’ lack of knowledge and training regarding how to work with students with ASD has been identified as a barrier the general educators face by other researchers, including Van Der Steen et al. (2020). As was mentioned in the findings, this was an issue the participants emphasized with statements such as, “I think that teachers who do not have any specialized background (in working with students with disabilities) are prepared at all” (Teacher 1). To help GE teachers be comfortable and successful in the teaching of students with ASD, teacher training programs should develop courses that allow their students to obtain more teaching experience with students with ASD (Park and Chitiyo, 2011). Thus, pre-service programs for GE teachers must develop courses that train teachers on how treat students with ASD in the GE environment. In addition to expanding pre-service training, professional development must be provided on an ongoing basis to GE teachers to improve their knowledge and confidence regarding teaching students with ASD. As has been stated in other research, “Very few teacher preparation programs offer more than 6-h training in EBPs” (Hsiao and Sorensen Petersen, 2019, p. 205). Such additional training has also found that the confidence and knowledge was higher in teachers working with students with ASD after they had completed effective trainings on fundamental ABA techniques (Leblanc et al., 2009). Given that teacher preparation programs have been found to be lacking in how they prepare (or fail to) teachers to work with students with ASD, teachers must have access to professional development that trains them in EBPs to support these students (Hsiao and Sorensen Petersen, 2019; Hasson et al., 2022).

Another theme identified in this study was the lack of collaboration opportunities with other educators and professionals who have expertise in special education and/or ASD. This issue was also noted during the observations, which revealed that general educators cannot provide the needed level of support to all students in the classroom, including those with ASD. In teacher training, the lack of collaboration could be overcome by creating opportunities for faculty from both special education and GE to work together to deliver the instruction to future teachers (Able et al., 2015; Majoko, 2019). By building relationships across disciplines, teacher educators can strengthen programs to support collaboration. This would allow for instructional strategies and accommodations for students with disabilities to be integrated into the pre-service GE curriculum. GE teachers would thereby be better able to maintain instruction and learning for all the students in the classroom through collaboration with other professionals with expertise in supporting students with disabilities such as ASD (Van Mieghem et al., 2020).

The last theme that emerged from the interviews was the need for resources. As Teacher 4 mentioned, the inability to meet the needs of students with ASD is often related to a lack of resources, especially in private programs. It is important for schools to provide the necessary resources to allow teachers to support all students, including those with disabilities, while also meeting the standards established for the provision of education to them (Wilson and Landa, 2019). The research found that teachers identify a number of resources (e.g., psychologists, therapists, and certain types of materials/equipment including technology) as necessary to the establishment of a successful inclusive classroom (Lindsay et al., 2013; Klibthong and Agbenyega, 2022; Leonard and Smyth, 2022). Furthermore, this research found that teachers encounter funding difficulties when it comes to the need for teaching resources and assistive technology devices for their students with ASD. This finding is supported by that of Hasson et al. (2022), who stated that students with ASD must receive the necessary funding to ensure they are well-educated (Lindsay et al., 2013; Hasson et al., 2022). In the case of private schools, such as that involved in this study, it is necessary that such programs seek funding from appropriate entities, such as state and federal government agencies, to ensure the availability of necessary resources for students with ASD.

### Conclusion, limitations, and future research

Students with ASD are now included in the GE setting in response to the requirements of legislation such as the Individuals with Disabilities Education Act [IDEA]., 2004. This qualitative research discussed the barriers the GE teachers face regarding teaching students with ASD in their GE classrooms. The researcher identified three themes and examined each in-depth. Based on the examination and analysis of these identified themes, this study found that GE teachers seek to be provided effective training, specialized equipment/technology, and opportunities to collaborate with their special education colleagues to properly support their students with ASD in their GE classrooms.

This study was limited by a few factors. For example, since case study methodology was employed, the researcher interviewed only four female teachers so as to acquire a deep understanding of the perceptions of the teachers. The duration of the interviews was also limited, only 30–45 min, due to the teachers’ availability. Had the interviews been longer, it is possible more data could have been obtained. Another limitation of the study is the setting, and private schools operate under different funding constraints and have different requirements under the law than do public schools, which impacts how teachers view the inclusion of students with ASD in the GE classroom. Future research should investigate what specific training for teachers of students with ASD would be most useful and effective in supporting teachers in creating an inclusive environment for students with ASD in the GE classroom.

## Data availability statement

The raw data supporting the conclusions of this article will be made available by the authors, without undue reservation.

## Ethics statement

The studies involving human participants were reviewed and approved by King Saud University. The patients/participants provided their written informed consent to participate in this study.

## Author contributions

MA contributed to the design and implementation of the research, as well as to the analysis of the results and to the writing of the manuscript. The author confirms being the sole contributor of this work and has approved it for publication.

## Appendix

### Appendix A

Text of general education teacher Participant interview questions.

• Can you tell me about your beliefs on inclusion?
• In your opinion, what are some of the pros and cons of inclusive school programs?
• In your opinion, do general education teachers have positive or negative attitudes toward inclusion of autism? If yes, what makes you say that? If no, how do you know?
• In your opinion, are general education environments ready to support students with ASD?
• Do you feel that you have the knowledge that allow you to use various accommodations and modifications to help adapt the environment for students with ASD in the general education classroom? If yes, can you explain please? If no, Why?
• Can you tell me about some barriers that you face when teaching students with ASD in your classroom?
